# High-precision, mass dependent Si isotope measurements *via* the critical mixture double-spiking technique

**DOI:** 10.1039/d4ja00152d

**Published:** 2024-08-30

**Authors:** Xiao-Ning Liu, Martijn Klaver, Remco C. Hin, Christopher D. Coath, Hong Chin Ng, Tim Elliott

**Affiliations:** a Bristol Isotope Group, School of Earth Science, University of Bristol Bristol BS8 1RJ UK xiaoning.liu@bristol.ac.uk; b Institute of Environmental Geology and Geoengineering, Consiglio Nazionale delle Ricerche (CNR) Italy

## Abstract

We have developed a new method for measuring mass dependent Si isotope fractionation *via* critical mixture double-spiking. Samples need to be spiked before column chemistry to guarantee full equilibrium between the sample and double-spike (^29^Si–^30^Si spike). An iterative addition of the double-spike to the sample, usually 2–4 times, is needed to generate a solution very close to the critically spiked mixture. We use a double-pass cyclonic quartz spray chamber, as it gives the highest signal-to-noise ratio. In conjunction with 6 μg ml^−1^ Si solution to yield intense Si isotope beams, this setup results in an ∼25 V (with 10^11^ Ω resistor) signal on ^28^Si^+^, while on-peak noise is less than 0.06 V. A typical sample analysis comprises 8 repeats (*n* = 8) of an individual sample measurement (for each repeat *n* = 1, 168 second analysis time) normalised to bracketing measurements of critically double-spiked NIST SRM 8546 (commonly known as NBS28). Each of these *n* = 8 analyses consumes about 13 μg of sample Si and yields a mean *δ*^30/28^Si with a precision of approximately ±0.03‰ (2 s.e., 2 × standard error of the mean). Over a 16 month period, the reproducibility of the 11 mean *δ*^30/28^Si values of such *n* = 8 analyses of the silicate reference material BHVO-2 is ±0.03‰ (2 s.d., 2 × standard deviation), which is 2 to 8 times better than the long-term reproducibility of traditional Si isotope measurement methods (∼±0.1‰, 2 s.d., *δ*^30/28^Si). This agreement between the long-term and short-term variability illustrates that the data sample the same population over the long and short terms, *i.e.*, there is no scatter on the timescale of 16 months additional to what we observe over twenty hours (the typical timescale in one analytical session). Thus, for any set of *n* repeats, including *n* >8, their 2 s.e. should prove a useful metric of the reproducibility of their mean. Three international geological reference materials and a Si isotope reference material, diatomite, were characterised *via* the critical mixture double-spiking technique. Our results, expressed as *δ*^30/28^Si_NBS28_, for BHVO-2 (−0.276 ± 0.011‰, 2 s.e., *n* = 94), BIR-1 (−0.321 ± 0.025‰, 2 s.e., *n* = 27), JP-1 (−0.273 ± 0.030‰, 2 s.e., *n* = 19) and diatomite (1.244 ± 0.025‰, 2 s.e., *n* = 20), are consistent with literature data, *i.e.*, within the error range, but much more precise.

## Abstract 2

We developed critical mixture double-spiking Si isotope measurement, achieving an enhanced long-term reproducibility of ±0.03‰ (*δ*^30^Si, 2 s.d.), which is ∼4 times better than the current methods' long-term reproducibility over ±0.1‰ (*δ*^30^Si, 2 s.d.).

## Graphical abstract

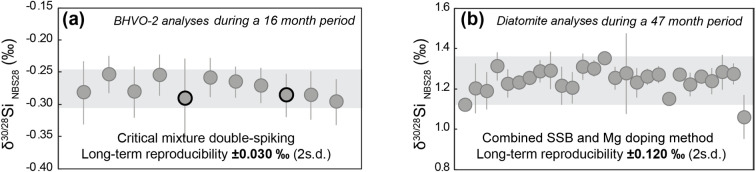

## 1 Introduction

The development of multi-collector inductively coupled plasma mass spectrometry (MC-ICPMS), combined with sample-calibrator bracketing (often colloquially referred to as “sample-standard bracketing”), both with or without Mg doping, has significantly improved the precision of Si isotope ratio measurements and promoted the development of the Si isotope systems (*e.g.*, ref. 1–7). The attainment of measurement precision in the range of *ca.* ±0.1‰ (ref. 8) on *δ*^30/28^Si has helped determine that the accessible mantle has a homogeneous Si isotope composition (−0.29 ± 0.07‰, ref. 2, 5, 8 and 9). This value is similar to lunar samples (−0.29 ± 0.08‰, ref. 10) but different from meteoritic materials (−0.3 to −0.8‰, ref. 3–5, 9 and 11). The discrepancy in Si isotope composition between primitive meteorites, that could represent Earth's building blocks, and the terrestrial mantle provides an important clue to the accretion and differentiation of the Earth. For instance, the non-chondritic Si isotope composition of the upper mantle might reflect isotopically light Si in the core,^5,12,13^ a hidden reservoir in the lower mantle,^14^ the result of heterogeneous accretion,^9^ the loss of light Si isotopes during the moon forming impact^3,4^ or by melt vaporisation in the planetesimal stage of accretion.^15^ Higher analytical precision may be helpful to resolve such contrasting models. For instance, a closer examination of oceanic island basalts with a lower mantle signature (*e.g.*, high ^3^He/^4^He) to determine whether they have anomalous Si isotope ratios potentially provides a test of the hypothesis of a residual reservoir from primordial magma ocean crystallisation.^14^

The current method used to correct Si isotope measurements for instrumental mass fractionation, sample-standard bracketing (sometimes in combination with Mg-doping), critically relies on identical mass fractionation in the MC-ICPMS during measurement of samples and the reference material (*e.g.*, ref. 16 and 17). Differences in the matrices of measured Si solutions can introduce biases in data measured in such a way. Several oxy-anion species, *e.g.*, SO_4_^2−^ and PO_4_^3−^, are co-eluted with Si in the typical chromatographic procedure used to purify Si and can be problematic for samples enriched in such elements. Such matrix effects can have a pronounced effect on instrumental mass fractionation. Double-spiking is the only method that provides robust correction for such fractionation during sample measurement.^18^

For these reasons we have developed a new double spike method for determination of mass dependent Si isotope variations. This technique has previously been successfully applied to Mg isotope analysis and achieved notable improvements in reproducibility (2 s.d., 2 × standard deviation, long-term reproducibility of ±0.027‰ for *δ*^26/24^Mg,^19,20^ compared to ±0.07‰, 2 s.d., with sample-standard bracketing^21^). Like Mg, Si has three naturally abundant isotopes and its isotopic composition cannot be measured *via* the conventional double spike technique,^18,22^ but the critical mixture method^23^ can be applied. In this paper, we report on the development of a critical mixture double-spiking method for Si isotope measurements that significantly improves the reproducibility of *δ*^30/28^Si analyses relative to the ±0.1‰ (2 s.d.) typical of sample standard bracketing analyses.^8^ Many aspects of analysing Si isotopes by critical mixture double-spiking are similar to conventional methods for analysing Si isotope compositions by MC-ICPMS, *e.g.*, ion exchange chromatography and MC-ICPMS set-up, so we mostly focus here on the aspects that are different to the conventional techniques and optimise the precision possible using this approach.

## 2 Experimental methods

### 2.1 Critical mixture double-spiking theory

**Fig. 1:**
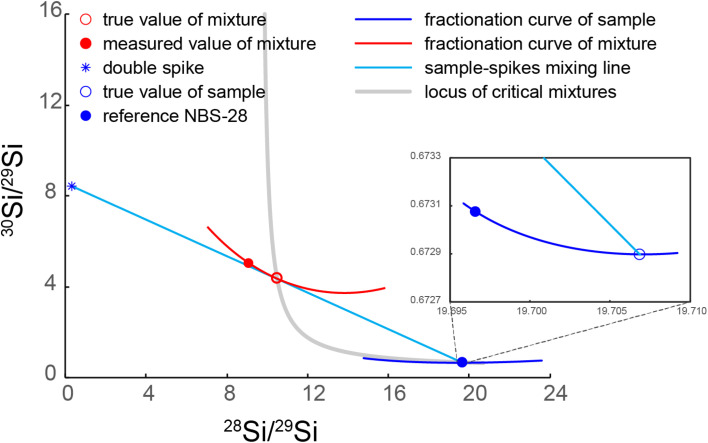
Illustration of critical mixture double-spiking with Si isotopes. The red open circle indicates the critically spiked mixture, whose fractionation curve (red line) is tangential to the sample–spike mixing line (cyan). The double-spike composition is from our calibrated result. NBS-28 composition is from Ding *et al.*^26^ and the sample illustrated in the figure has a *δ*^30/28^Si value of −0.03‰ normalised to reference NBS-28.

The double spike technique enables accurate correction for mass-dependent isotope fractionation during sample purification and measurement.^18,23,24^ Its application has long been restricted to the analysis of elements with at least four isotopes owing to mathematical inversion requirements,^16,18,22^ leaving three key major elements (Si, Mg, and K), which only have three stable (or long-lived) isotopes, excluded from this approach. An expansion of the double spike technique for the three-isotope systems was expounded by Coath *et al.*^23^ This work was built on the notion of critical mixtures discussed by Hofmann^25^ and so it was termed critical mixture double-spiking. In brief, the critical mixture refers to the exact point on the mixing line between the double-spike and sample where the instrumental mass fractionation line of the mixture is tangential to the mixing line, *i.e.*, d*α*/d*β* = 0 (ref. 23) (Fig. 1), where *α* is the sample mass-fractionation relative to the reference and *β* is the instrumental mass-bias. This means that, whenever the sample is critically spiked, any small deviation in the estimation of *β* (obtained, for instance, through sample-standard bracketing) does not propagate into the inaccuracy on *α*. Thus, the instrumental mass bias *β* can be pre-determined, meaning that the number of independent isotope ratios required to solve the double spike equations for *α* decreases from three to two, and so *α* can be determined using the two independently determined isotope ratios in a three-isotope system. In practice, however, the exact critical mixture between the sample and double-spike is very hard to make. Therefore, we treat a solution that is within ±0.5% (molar proportion) of the critical mixture as critically mixed. The effect of such small ±0.5% deviations from the critical has been shown not to compromise accuracy at the ±0.01‰ level to yield accurate results.^15,23^

### 2.2 ^29^Si–^30^Si double-spike preparation

Single spikes of ^29^Si and ^30^Si were purchased from the Oak Ridge National Laboratory. These single spikes were in the form of SiO_2_ and digested in 15 ml PFA vials in ∼1 ml 30% NaOH solution (Suprapur ®, Si concentration less than 1 μg g^−1^) for 72 h at 180 °C. After digestion, 18.2 MΩ cm Milli-Q (M.Q.) water was added to create concentrated solutions of Si in aqueous NaOH, which were purified over the cation-exchange columns described in Section 2.5 to eliminate contamination of metals likely originating from the spike containers. After purification, the single spikes were diluted further and calibrated against the known isotope composition of Si reference material National Institute of Standards and Technology NIST SRM 8546 (often referred to, and likewise hereafter, by its historic name “NBS-28”)^26^ for their isotope compositions and concentrations. Subsequently, single spike aliquots were carefully mixed to make the optimal double-spike composition recommended by Coath *et al.*,^23^ described in section 4 ‘*Precision*’ of Coath *et al.*^23^ Then the double-spike was calibrated against NBS-28 (ref. 26) multiple times, following the steps described in section 7 ‘*Double-spike calibration*’ of Coath *et al.*^23^. The calibrated double spike has an isotope composition of 3.0817 (^29^Si/^28^Si) and 26.1352 (^30^Si/^28^Si) when calibrated against the NBS-28 reference values of ^29^Si/^28^Si and ^30^Si/^28^Si of 0.0507 and 0.0341 from Ding *et al.*^26^ (Fig. 1). With these compositions, the critical mixture parameter (*p*_c_, the molar proportion of double spike in a critical mixture) is 0.2936, calculated from eqn (14) & (15) of Coath *et al.*^23^

### 2.3 Silicate sample digestion

Digestion of rock samples follows the NaOH fusion protocols in Georg *et al.*^1^ About 5 to 10 mg of sample is added together with a ∼200 mg NaOH pellet (from EMSURE^®^, Si concentration less than 5 μg g^−1^) in a 7 ml lidded Ag crucible, and the mixture is fused in a pre-heated (730 °C) furnace for 10 minutes. After cooling, the fusion cake is dissolved in ∼1 ml M.Q. water and transferred to a 50 ml pre-cleaned Savillex centrifuge tube. Before sample transfer, the Ag crucible with sample solution is placed in a capped, flat base, cleaned 15 ml Savillex Teflon beaker and it is then sonicated three times (15 minutes for each). The crucible is subsequently rinsed 6 times with ∼0.5 ml M.Q. water, which is added to the sample solution, to ascertain complete sample recovery.

We also explored a ‘cold fusion’ sample digestion method following previous research.^27^ The sample and NaOH powder (ground EMSURE^®^ NaOH pellet) are mixed in a capped clean 7 ml Savillex PFA beaker together with ∼50 μl M.Q. water to help mix the sample with NaOH evenly. Then the mixture is left on a 200 °C hotplate for at least one week to achieve complete digestion. After digestion, the fusion cake is also dissolved in M.Q. water. This method is particularly suitable for small samples (less than 1 mg) due to the much lower Si blank, usually less than 10 ng Si, compared to the ∼1 μg Si blank in the digestion protocol with Ag crucible fusion above.

### 2.4 Preparing sample–spike mixtures

**Fig. 2:**
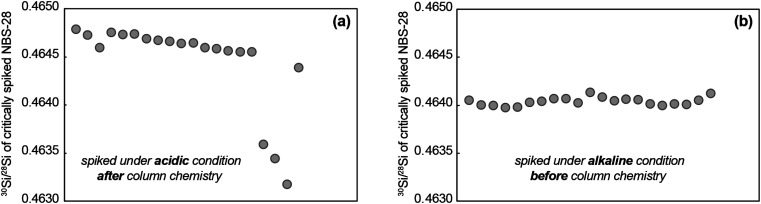
A comparison of the ^30^Si/^28^Si variations in a typical 24 hour sequence measurement by MC-ICPMS for critically spiked NBS-28 under different conditions. (a) Critically spiked under acidic conditions after ion exchange chromatography, the 2 s.d. of the 20 ratios is 9 × 10^−4^; (b) critically spiked under alkaline conditions before ion exchange chromatography, the 2 s.d. of the 20 ratios is 7 × 10^−5^. Both mixtures were heated on a 100 °C hotplate overnight after spiking and their Si concentration and acid molarity are the same. The measurements in panel (A) and (B) were made in two separate measurement sessions but the instrument setup and sensitivity were identical and magnet stability was normal during both sessions. The slight difference in the measured ratios is due to the different magnitude of instrumental mass bias from session to session.

Unlike critical mixture double-spiking applied to Mg isotope analysis,^15,19,28^ samples for Si isotope analysis are spiked before ion exchange chromatography because of the chemical behaviour of Si. In a high pH environment, the prevalent Si species is a stable anion, mainly SiO_2_(OH)_2_^2−^, while at low pH conditions, it forms a series of neutral species such as H_4_SiO_4_ and H_2_SiO_3_.^29^ In such a solution, the neutral Si species are likely colloidal^29^ and it is hard to reach a true equilibrium among such species.^30^ As the double spike technique requires complete isotopic equilibrium between the sample and spike, samples need to be spiked under alkaline conditions. A comparison of NBS-28 spiked before column purification under alkaline conditions and after column purification under acidic conditions is shown in Fig. 2. Both were heated on a 100 °C hotplate overnight. It is clear that the ^30^Si/^28^Si in the mixture spiked under alkaline conditions is more constant than in the mixture spiked under acidic conditions in a typical 24 hour-long measurement session (Fig. 2). Although the pronounced ^30^Si/^28^Si variation seen in the mixture spiked under acidic conditions may be related to the unstable instrumental mass bias rather than sample–spike disequilibrium, such significant drift in ^30^Si/^28^Si was frequently observed in samples spiked under acidic conditions but never seen in the samples spiked under alkaline conditions. Therefore, all the samples in this study are spiked in an alkaline environment before column chemistry to guarantee complete isotopic equilibrium between the sample and double-spike.

As the samples are spiked before ion exchange chromatography, the dissolved samples and double-spike are stored in 0.1 mol L^−1^ NaOH solution before mixing. Both sample and double-spike have a Si concentration of ∼50 μg ml^−1^. Aliquots of the sample solution (∼1.2 ml) and double-spike (∼0.5 ml) are mixed in a capped 5 ml Savillex autosampler vial. The weights of the sample solution and double-spike are carefully measured on a four-figure balance and these numbers are used later to adjust the spike proportion. Then the mixture is put on a 100 °C hotplate overnight to reach equilibrium. A ∼0.150 ml fraction of the mixture, also carefully weighed, with a concentration of ∼50 μg ml^−1^ Si, was acidified and purified through the column chemistry described in section 2.5. This purified fraction was then measured on the MC-ICPMS (see Section 2.6) to check the sample–spike mixing proportion. If the mixing proportion deviated more than 0.5% from criticality, the remaining unpurified mixture was adjusted by adding either more unspiked sample solution or double-spike to meet the critical mixing proportion. Then the adjusted mixture was heated overnight for spike–sample equilibration, and another 0.150 ml mixture fraction was taken, purified, and measured. This procedure was repeated until the mixture was within the bound of ±0.5% molar proportion from the critical mixture point (*p*_c_ of 0.2936, Section 2.2). Usually, two to four iterations are required to meet the ±0.5% bound. After being critically mixed, the whole solution is purified by ion exchange chromatography. The equations behind these iteration for obtaining critical mixtures are described in section 6 ‘*Preparing critical mixtures*’ of Coath *et al.*^23^

### 2.5 Ion exchange chromatography for purifying Si

The Si purification follows the ion-chromatography protocol with Bio-Rad AG50W-X12 cationic exchange resin developed by Georg *et al.*^1^ In brief, the sample solution is acidified to a pH of 2.3 (∼0.005 mol L^−1^ H^+^) to neutralise the Si species before loading onto pre-cleaned resin. The prevailing electrically neutral Si species (*e.g.*, H_4_SiO_4_, H_2_SiO_3_) and a small amount of anionic H_3_SiO_4_^−^ (in equilibrium with H_4_SiO_4_)^29^ barely interact with the cationic resin and can be directly collected when eluting with M.Q. water, while most other ions are adsorbed onto the cationic resin. In practice, 1 ml sample solution with 50 μg ml^−1^ Si is loaded and the Si is recovered in the following 3 ml M.Q. water eluant. Another 1 ml M.Q. water eluant (referred to as the ‘split’) is also collected after Si recovery to monitor the column yield, by assuming all the silicon is eluted. From the tiny amount of Si in this split, the column yield is over 99.98% for all our samples.

### 2.6 Instrumental analysis

Si isotope composition measurements were performed on a Thermo-Finnigan Neptune (serial no. 1020) MC-ICPMS under medium resolution mode (defined by *m*/Δ*m* ≥ 4000) in the Bristol Isotope Group, University of Bristol.

#### 2.6.1 The selection of sample introduction system

**Fig. 3:**
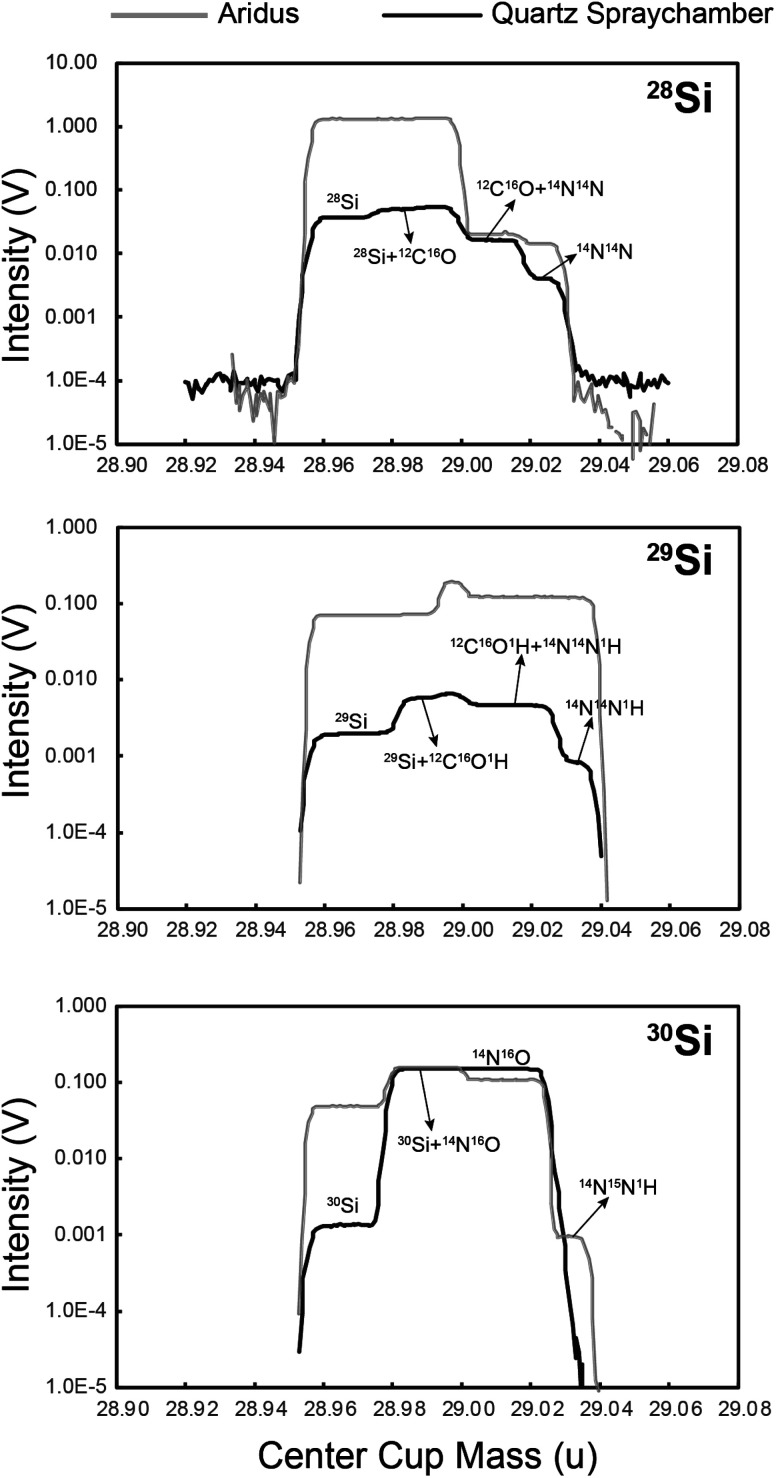
Mass scans containing the ^28^Si, ^29^Si and ^30^Si peaks and interfering species using different introduction systems in blank solution (0.15 mol L^−1^ HCl) at medium resolution (*m*/Δ*m* ≥ 4000, 5–95% peak height definition). Amplifiers with 10^11^ Ω feedback resistors were used. Grey line: samples introduced under dry plasma conditions using the Aridus. Black line: wet plasma sample delivery using a cyclonic, double pass, quartz spray chamber. Note the intensity is plotted on a log scale.

Three sample introduction systems, Aridus (Cetac Instrument), Apex IR (Elemental Scientific Inc.) and a cyclonic double pass quartz spray chamber, combined with a Savillex PFA nebuliser were investigated. Mass scans of the Si ion beams in a blank solution (0.15 mol L^−1^ HCl) are shown in Fig. 3. Since the Apex yielded a similar sensitivity and interference species to the Aridus, we only show the mass scan of Aridus in Fig. 3.

**Fig. 4:**
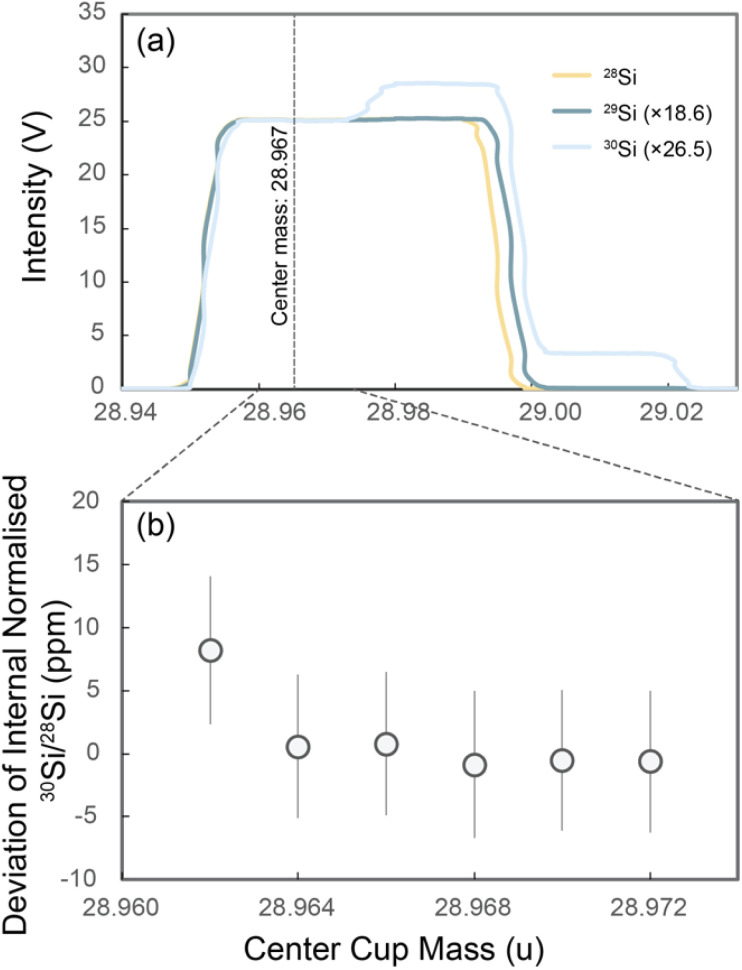
(a) Mass scan of Si isotopes in a 6 μg ml^−1^ unspiked NSB-28 Si solution (in 0.15 mol L^−1^ HCl) in medium resolution (*m*/Δ*m* ≥ 4000); the measurement position of the centre cup mass is on the peak shoulder at 28.967 u. (b) Parts per million (ppm) deviation of the internally normalised ^30^Si/^28^Si for the mass range from 28.962 to 28.972 u of the centre cup mass.

With the Aridus as the introduction system, the N_2_ gas flow is switched off to reduce the ^14^N^16^O interference. A solution of 1 μg ml^−1^ Si yielded a ^28^Si^+^ beam current of ∼15 V on default 10^11^ Ω amplifiers, while the intensity of ^28^Si^+^ in the 0.15 mol L^−1^ HCl wash solution could be as high as ∼1 V (Fig. 3). For comparison, 1 μg ml^−1^ Si introduced with the quartz spray chamber yielded ∼4 V on ^28^Si^+^ (Fig. 4a) but only <0.06 V on ^28^Si^+^ in the wash solution. The Aridus thus yields an overall better sensitivity, but at the cost of a lower signal/noise ratio. As to interferences, the Aridus introduction system shows different (some lower) relative intensities of molecular interference species to Si in the blank solution compared with the quartz spray chamber, *e.g.*, the obvious ^12^C^16^O^+^ and ^12^C^16^O^1^H^+^ interferences seen in the mass scan with the quartz spray chamber are less prominent when using the Aridus (Fig. 3). Nevertheless, the intensities of interfering species with the Aridus are generally higher due to its higher sensitivity. Considering Si is a major element and measurements are rarely limited by the amount of sample, Si beam intensity can be easily increased with a higher analyte concentration and we selected the quartz spray chamber as the introduction system because of its much higher signal/noise ratio and lower interference intensities.

#### 2.6.2 Peak shoulder scans of Si isotope profiles

As shown in Fig. 3 and 4a, measurements cannot be made in the middle of the Si peaks because of the presence of molecular interferences. However, careful cup alignment and measurement offset to lower mass allow complete resolution of these interferences (“measuring on the peak-shoulder”). The flatness of the left shoulder of the Si peaks was tested to assess the possible influence of small drifts of the magnetic field during a long measurement session (Fig. 4b). Each datapoint in Fig. 4b consists of 100 analysis cycles with 8.4 s integration time for each cycle. The deviations of the internally normalised ^30^Si/^28^Si for these points are within ±5 parts per million (ppm) of 0 in the mass range of 28.964 to 28.972 (u, center cup mass) (Fig. 4b). Therefore, mass-independent effects from variable tailing of interfering species are less than ±5 ppm in this mass range.

#### 2.6.3 Sample measurements by MC-ICPMS

A combination of platinum Jet cone and nickel H skimmer cone was used. We initially used the platinum cone as it is more resistant to the HCl corrosion and retained this for consistency, but nickel cones can also yield reliable data. The sample solution is in 0.15 M HCl with a concentration of 6 μg ml^−1^ Si. The solution is aspirated into the instrument through a double-pass cyclonic quartz spray chamber and a Savillex PFA nebuliser (∼100 μl min^−1^ uptake rate). An intensity of ∼20 to 30 V was obtained on ^28^Si (un-spiked sample, Fig. 4a), while the instrumental blank of the ^28^Si intensity is less than 0.06 V. Feedback amplifiers with 10^11^ Ω resistors were used to measure the signals of all three Si isotope beams. One measurement consists of 20 cycles with 8.4 second integration time for each cycle and consumes ∼1.68 μg Si (sample plus double-spike). Each sample analysis comprised six to nine times (*n*) such measurements and the reported *δ*^30/28^Si is the mean value of the repeats. Three measurements of the un-spiked reference NBS-28 are performed at the beginning, after one-third and two-thirds of the sequence, and at the end of the sequence to provide constraints on the instrumental mass bias *β*, which is determined as *β* = −ln(*M*/*m*)/*P*_*i*_, where *M* is the true isotope ratio (from ref. 26), *m* is the measured isotope ratio, and *P*_*i*_ is the natural logarithm of the isotope mass ratio. The *β* for each individual sample is then estimated *via* interpolation. A bracketing measurement of critically spiked reference NBS-28 is performed every three sample measurements to correct any non-exponential component in the instrument mass bias. The uncertainties reported on *δ*^30/28^Si are two times the standard error of the mean (2 s.e). The 2 s.e. is calculated from a standard deviation (s.d.) (2 s.e. = 2 s.d./√*n*, where *n* is the number of repeat measurements), which is determined by a homoscedastic approach by pooling over all the standards and samples in each analytical session.^31^ The detailed data reduction process can be found in the Appendix.

## 3 Results and discussion

### 3.1 Measurements with a sample-standard bracketing method

As an initial test of the reliability of our column chemistry and mass measurement position, we performed sample-standard bracketing analyses of three international rock standards (Hawaiian basalt BHVO-2, Icelandic basalt BIR-1, and peridotite JP-1) with the same but spike-free settings we described in Section 2.6.3, *i.e.*, the combination of Jet sample cone and H skimmer cone, double-pass cyclonic quartz spray chamber and a Savillex PFA nebuliser. The silicon isotope intensity is measured on a shoulder position of 28.967 (Fig. 4). The results are shown in Table 1. Our measurements of these rock standards are consistent with the values reported in the literature,^2,32,33^*i.e.*, within the error range. In Table 1 we further report values of *Δ*^30^Si (the relative deviation of ^30^Si/^28^Si, exponentially internally normalised to ^29^Si/^28^Si, from the internally normalised NBS28 standard) which are all within error of zero. This demonstrates the measurements are free from any significant interfering species.

**Table 1:** Si isotope composition of geological reference materials measured *via* the sample-standard bracketing method^a^

	*δ* ^29/28^Si (‰)	2 s.d. (‰)	*δ* ^30/28^Si (‰)	2 s.d. (‰)	*Δ* ^30^Si (ppm)	2 s.e. (ppm)	*n*	Literature *δ*^30/28^Si ± 2 s.d.^b^(‰)
BHVO-2	−0.143	0.047	−0.282	0.091	−1	24	9	−0.27 ± 0.1 (ref. 2), −0.27 ± 0.08 (ref. 32)
BIR-1	−0.169	0.086	−0.331	0.157	1	16	9	−0.27 ± 0.07 (ref. 32), −0.33 ± 0.13 (ref. 33)
JP-1	−0.142	0.050	−0.282	0.080	−3	18	9	−0.29 ± 0.08 (ref. 32)

a *Δ* ^30^Si = *δ*^30/28^Si − *δ*^29/28^Si/*β*, where *β* = ln[(*m*_29_/*m*_28_)/(*m*_30_/*m*_28_)],^34^*m*_*i*_ is the mass of silicon isotope *i*. Both the sample and bracketing standard are thus internally normalised in *Δ*^30^Si values.

b We only presented two widely cited literature BHVO-2 *δ*^30/28^Si values from ref. 2 and 32. The remaining literature BHVO-2 *δ*^30/28^Si values are in the error range with the presented two literature values and our data, and are illustrated in Fig. 6.

We report precision in two different ways (in Table 1 and elsewhere) according to the style of measurement. For sample-standard bracketing, drift in plasma conditions within and between sessions is imperfectly corrected. This is a result of the different mass bias responses of samples, with residual matrices, and pure bracketing standards to these changes in plasma conditions.^35,36^ This differential response imparts a significant component of measurement variability. In order to account for this uncertainty, the precision of a sample-standard bracketing measurement is conventionally characterised by the 2 s.d. of the individual analyses from which it is comprised,^17,32,37^ as further discussed in section 3.2.

For internally normalised measurements, such as *Δ*^30^Si (Table 1) or double spiked analyses (Table 2), internal mass-bias normalisation naturally corrects for differences in mass bias between the sample and bracketing standards, resulting in measurements whose precisions are potentially, dominantly counting statistics limited. We therefore report precisions of internally normalised measurements as the 2 s.e. of constituent analyses, which is also conventional and justified in section 3.2 specifically for our critical mixture double-spiking method.

**Table 2:** Si isotope compositions of reference materials measured via the critical mixture double-spiking method. Each row represents the mean of a single digestion of a reference material. All repeated measurements of the same digestion occurred in one analytical session where *n* is the number of these repeats

Standards	*δ* ^30/28^Si	Pooled 2 s.e.	*n*
**Hawaiian basalt BHVO-2**			
	−0.282	0.049	9
	−0.254	0.029	6
	−0.281	0.040	6
	−0.255	0.032	8
a	−0.291	0.062	8
	−0.259	0.031	7
	−0.265	0.024	10
	−0.271	0.027	10
a	−0.286	0.034	10
	−0.286	0.038	10
	−0.296	0.035	10
**Mean**	**−0.276**	**0.011**	**94**

**Icelandic basalt BIR-1**			
	−0.291	0.049	9
	−0.332	0.045	8
	−0.340	0.035	10
**Mean**	**−0.321**	**0.025**	**27**

**Peridotite JP-1**			
	−0.258	0.049	9
	−0.286	0.035	10
**Mean**	**−0.273**	**0.030**	**19**

**Diatomite**			
	1.244	0.035	10
	1.244	0.035	10
**Mean**	**1.244**	**0.025**	**20**

a Sample was digested *via* ‘cold fusion’.

### 3.2 Long-term reproducibility

Long-term reproducibility of analyses investigating mass-dependent isotope fractionation is commonly determined by repeated measurements of an international reference material over long periods of time and determining the 2 s.d. over all those individual repeats. As described above, owing to the common use of sample-standard bracketing analysis techniques, this long-term reproducibility (or, alternatively, the 2 s.d. of individual repeats of a single sample) is typically considered the best estimate of the uncertainty of the mean of a sample analysed multiple times. This approach to uncertainty inherently implies that the mean of multiple repeated analyses of a sample cannot be determined to higher precision than the mean of a single analysis of that sample. As we will confirm below, this notion is correct for sample-standard bracketing analyses, rendering a 2 s.d. of all individual analyses of a reference material over a long period of time a good measure of long-term reproducibility. However, as we will also show below, these analytical, statistical limitations do not apply to (critical mixture) double-spiked analysis as the mean of samples analysed with this method can be determined to higher precision through multiple repeats. A long-term reproducibility determined on all individual repeats is thus not a good measure of the uncertainty of the mean of repeated sample analyses with this method. We therefore propose here that the 2 s.d. on the measurement session means of a reference material (6–8 repeats in a single measurement session) taken from various measurement sessions over a long period of time is a better measure of the long-term reproducibility of our critically mixed double-spike analyses. We determine the long-term reproducibility in this manner for data obtained from both critical sample double-spiking and sample-standard bracketing to quantify the improvement in precision of our new technique to the conventional one.

We use multiple measurements of the international rock standard BHVO-2 to evaluate the long-term reproducibility of our critical mixture double-spiking method. Two samples of BHVO-2 (indicated with in Table 2) have been dissolved using the ‘cold fusion’ method with a smaller amount of sample (∼0.5 mg BHVO-2 powder). Their *δ*^30/28^Si values show no statistically significant differences at 2 s.d. with the samples dissolved with high temperature NaOH fusion.

**Fig. 5:**
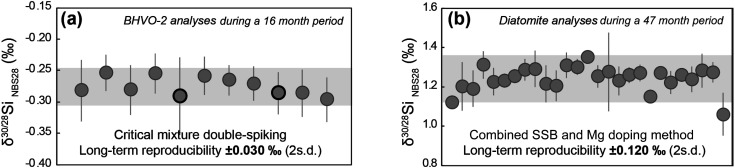
(a) BHVO-2 measurements *via* critical mixture double-spiking in 11 analytical sessions during a 16 month period. Each symbol represents the mean, with 2 s.e. uncertainty, of 6–9 repeated measurements in a single session. The grey bar indicates the 2 s.d. long-term reproducibility. The two highlighted datapoints are the samples dissolved using the ‘cold fusion’ method. (b) Diatomite analyses *via* sample-standard bracketing combined with Mg doping in 27 analytical sessions over a 47 month period, performed by Hong Chin Ng (Ng *et al.*^38^) at Bristol. Each point represents the mean and its 2 s.e in each individual analytical session. The grey bar is the 2 s.d. of the means of all the 27 analytical sessions.

As shown in Fig. 5a and Table 2, the 11 means of BHVO-2 measurements obtained in 11 analytical sessions during a 16 month period overlapped within their individual 2 s.e. uncertainties. The overall dataset has a variability of ±0.030‰ (2 s.d.). Notably, this 2 s.d. long-term reproducibility is very similar to the individual 2 s.e. measurement precisions (±0.03 to ±0.04‰) (Table 2). These observations imply a dataset drawn from a single population. While we demonstrate that the 2 s.e. of individual measurements is a robust measure of sample reproducibility only for measurements comprising about 8 repeats, the 2 s.e. of samples measured with a larger number of repeats, to greater precision, should also yield a reasonable estimate of intermediate precision. For a major element such as Si, where measurements are rarely sample limited, this is an important consideration. Hence we report the 2 s.e. on the means of the rock standard data in Table 3 as a useful bound on reproducibility. We have reached similar conclusions in our previous assessment of the uncertainties of critical mixture double-spiking analysis for Mg isotope ratios.^28^

**Table 3:** Comparison of the long-term reproducibility for Si isotope ratios analysed by critical mixture double-spiking and obtained by alternative methods presented in the literature

Long-term reproducibility (± 2 s.d. ‰ on *δ*^30/28^Si)	*n* (repeats)	*N* (analytical sessions)	Time period (in months)	Typical 2 s.d. (‰) of individual sample repeats	Typical 2 s.e. (‰) of individual sample repeats (*n* = 8)	Method	Ref.
±0.030	94	11	16	±0.090	±0.030	Critical mixture double spike	This study
±0.120	92	27	47	±0.100	±0.030	Sample-standard bracketing combined with Mg doping	Ng *et al.*^38,39^
±0.140	Over 300	16	10	±0.100	±0.030	Sample-standard bracketing	Georg *et al.*^1^
±0.080	42	5	12	±0.100	±0.030	Sample-standard bracketing combined with Mg doping	Zambardi and Poitrasson^32^
±0.100	257	20	12	±0.100	±0.030	Sample-standard bracketing	Savage *et al.*^2^
±0.250	Over 200	40	Over 20	±0.150	±0.040	Sample-standard bracketing	Chakrabarti and Jacobsen^40^
±0.150	400	—	18	±0.120	±0.040	Sample-standard bracketing	Armytage *et al.*^5^
±0.120	76	13	—	±0.080	±0.020	Sample-standard bracketing	Pringle *et al.*^6^

We can compare our critical mixture double-spiking results with the long-term monitoring of the Si isotope composition of the standard material diatomite, measurements made at Bristol using sample-standard bracketing combined with Mg doping^38,39^ (Table 3 and Fig. 5b). When pooling the session means of these diatomite analyses, the long-term reproducibility (2 s.d.) based on 27 analytical sessions over 47 months is ±0.120‰ (ref. 38 and 39) (Fig. 5b). Such ±0.120‰ long-term 2 s.d. reproducibility is comparable to the individual 2 s.d. uncertainty (∼±0.100‰) within an analytical session, but obviously greater than the individual 2 s.e. uncertainty of means determined from repeated analysis within each session (Fig. 5b and Table 3). Table 3 further shows that this systematics holds in general for sample-standard bracketing studies in the literature. Namely, the long-term reproducibility of previous Si isotope composition measurements is ∼0.1 to ∼0.15‰, similar to the 2 s.d. typically obtained on repeated sample analyses in a single analytical session and so markedly higher than the associated 2 s.e. of the mean of ∼8 repeats (∼±0.03‰). In terms of a useful metric for intermediate reproducibility, these observations validate both our use of the 2 s.e. of the mean with one analytical session in the case of critical mixture double-spiking, and the more conservative use of session 2 s.d. in the case of sample–standard bracketing, as widely used in the literature.

### 3.3 Reference material measurements *via* critical mixture double-spiking

**Fig. 6:**
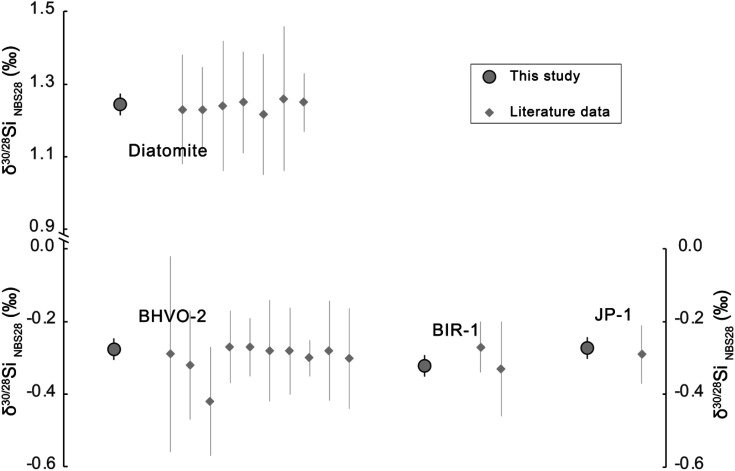
Si isotope ratio of reference materials measured *via* critical mixture double-spiking compared to literature data. Literature data are from ref. 2, 5, 7, 9, 32, 33, 38, 40–44. Note that the uncertainties reported are the 2 s.d^32^ long-term measurement reproducibility.

Together with BHVO-2, two other international geological reference materials (BIR-1 and JP-1) and a diatomite Si isotope reference material were measured by the critical mixture double-spiking technique multiple times over 16 months (Table 2). A comparison of the measurements of these reference materials by critical mixture double-spiking and literature data is shown in Fig. 6. All the values obtained *via* critical mixture double-spiking agree well with the literature data, but the former show much smaller uncertainties. As a conservative estimate of reproducibility, Fig. 6 illustrates the long-term 2 s.d. for critical mixture double spiking taken from our repeat measurements of BHVO-2 (section 3.2). However, the 2 s.e. of the more intensively analysed BHVO-2 (Table 2) is likely representative of its reproducibility.

## 4 Conclusions

Critical mixture double-spiking can produce Si isotope compositions that are accurate and precise. The long-term reproducibility of the method over 16 months based on BHVO-2 analyses (in 11 dissolutions and an equal number of analytical sessions) is ±0.030‰ on *δ*^30/28^Si (2 s.d.), which is 2 to 8 times better than the sample-standard bracketing Si isotope measurement methods presented in the literature (*e.g.*, ref. 2), with and without Mg-doping. Our mean *δ*^30/28^Si results for international geological reference materials and a diatomite Si isotope reference agree well, *i.e.*, within the error range, with literature data (BHVO-2 = −0.276 ± 0.011‰; BIR-1 = −0.321 ± 0.025‰; JP-1 = −0.273 ± 0.030‰; and diatomite = 1.244 ± 0.025‰, all uncertainties 2 s.e.) but have substantially smaller uncertainties owing to our new analytical technique. We show that measurement uncertainties of our critical mixture double spiking approach have negligible additional scatter over the 16 month period and so reported 2 s.e. provide a reliable indicator of sample reproducibility.

## Author contributions

Xiao-Ning Liu: conceptualisation of this project, investigation and developing the method, data analysis and writing the (original draft) manuscript. Martijn Klaver: conceptualisation of this project; investigation and developing the method and writing (review & editing). Remco C. Hin: conceptualisation of this project, funding acquisition and writing (review & editing). Christopher D. Coath: conceptualisation of this project, investigation and developing the method and writing (review & editing). Hong Chin Ng: investigation and developing the method and data analysis. Tim Elliott: conceptualisation of this project, funding acquisition, supervising the project and writing (review & editing).

## Conflicts of interest

There are no conflicts to declare.

## A Appendix

The protocols of the critical mixture double spike Si isotope analysis method.

(1) Mixing the sample and double-spike

The carefully weighed sample solution and Si double-spike (^29^Si–^30^Si spike) are mixed under alkaline conditions and the mixture is left on a 100 °C hotplate overnight to reach isotope equilibrium.

(2) Adjusting the mixing proportion to the critical mixture point

A 0.15 ml aliquot of the mixture is taken and purified with column chemistry to remove all the matrix elements. Then the purified Si solution is analysed on the instrument together with the reference material NBS-28. The analysis of the NBS-28 provides a reference for the instrument mass bias *β*, *β* = −ln(*M*/*m*)/*P*_*i*_, where *M* is the true isotope ratio (from ref. 26), *m* is the measured isotope ratio, and *P*_*i*_ is the natural logarithm of the isotope mass ratio. Then the *β* for each individual sample is estimated *via* interpolation. With *β* as an input, one can solve the double spike equations (see ref. 22) to get the value of *p* (the molar proportion of double spike in the mixture). Then comparing the *p* with *p*_c_ (0.2936, the molar proportion of the double spike in a critical mixture). If *p* > *p*_c_, the mixture is over-spiked and more sample needs to be added, and if *p* < *p*_c_, the mixture is under-spiked and more double-spike is required. The adjustment of the amount of the sample or double-spike is explained in section 6 of Coath *et al.*^23^ Step (2) is repeated until the difference between *p* < *p*_c_ is less than 0.5%.

(3) Sample analysis

The critically mixed samples from step (2) are purified and analysed on a Neptune MC-ICPMS. NSB-28 measurements are also performed to provide the reference of instrument mass bias *β.* The *β* for each individual sample is also estimated *via* the interpolation method. We also performed critically spiked NBS-28 to normalise any non-exponential instrumental fractionation. With the instrument mass bias *β* as input, one can solve the double spike equations (see ref. 22) to get the value of sample mass-dependent fractionation parameter *α*, relative to the reference material NBS-28. Then the *α* value of each individual sample is normalised to the *α* value of the critically spiked NBS-28 to obtain a normalised *α*_N._ The commonly used *δ* notation is then obtained from *δ* = e^−*α*_N_×*P*_*i*_^ − 1, where *P*_*i*_ is the natural logarithm of the isotope mass ratio.

## References

[cit1] Georg R., Reynolds B. C., Frank M., Halliday A. N. (2006). Chem. Geol..

[cit2] Savage P., Georg R., Armytage R., Williams H., Halliday A. (2010). Earth Planet. Sci. Lett..

[cit3] Zambardi T., Poitrasson F., Corgne A., Méheut M., Quitté G., Anand M. (2013). Geochim. Cosmochim. Acta.

[cit4] Fitoussi C., Bourdon B. (2012). Science.

[cit5] Armytage R., Georg R., Savage P., Williams H., Halliday A. (2011). Geochim. Cosmochim. Acta.

[cit6] Pringle E. A., Moynier F., Savage P. S., Jackson M. G., Moreira M., Day J. M. (2016). Geochim. Cosmochim. Acta.

[cit7] Yu H.-M., Li Y.-H., Gao Y.-J., Huang J., Huang F. (2018). Chem. Geol..

[cit8] Savage P. S., Armytage R. M., Georg R. B., Halliday A. N. (2014). Lithos.

[cit9] Fitoussi C., Bourdon B., Kleine T., Oberli F., Reynolds B. C. (2009). Earth Planet. Sci. Lett..

[cit10] Armytage R., Georg R., Williams H., Halliday A. (2012). Geochim. Cosmochim. Acta.

[cit11] Savage P. S., Moynier F. (2013). Earth Planet. Sci. Lett..

[cit12] Georg R. B., Halliday A. N., Schauble E. A., Reynolds B. C. (2007). Nature.

[cit13] Ziegler K., Young E. D., Schauble E. A., Wasson J. T. (2010). Earth Planet. Sci. Lett..

[cit14] Huang F., Wu Z., Huang S., Wu F. (2014). Geochim. Cosmochim. Acta.

[cit15] Hin R. C., Coath C. D., Carter P. J., Nimmo F., Lai Y. J., Pogge von Strandmann P. A. E., Willbold M., Leinhardt Z. M., Walter M. J., Elliott T. (2017). Nature.

[cit16] Klaver M., Coath C. D. (2019). Geostand. Geoanal. Res..

[cit17] Teng F. Z., Yang W. (2014). Rapid Commun. Mass Spectrom..

[cit18] Albarède F., Beard B. (2004). Rev. Mineral. Geochem..

[cit19] Liu X., Hin R. C., Coath C. D., van Soest M., Melekhova E., Elliott T. (2022). Geochem. Perspect. Lett..

[cit20] He Y., Sun A.-Y., Zhang Y.-C., Yang R.-Y., Ke S., Wang Y., Teng F.-Z. (2022). Solid Earth Sci..

[cit21] Teng F.-Z., Li W.-Y., Ke S., Marty B., Dauphas N., Huang S., Wu F.-Y., Pourmand A. (2010). Geochim. Cosmochim. Acta.

[cit22] Rudge J. F., Reynolds B. C., Bourdon B. (2009). Chem. Geol..

[cit23] Coath C. D., Elliott T., Hin R. C. (2017). Chem. Geol..

[cit24] Dodson M. (1963). J. Sci. Instrum..

[cit25] Hofmann A. (1971). Earth Planet. Sci. Lett..

[cit26] Ding T., Wan D., Bai R., Zhang Z., Shen Y., Meng R. (2005). Geochem. Cosmochim. Acta.

[cit27] Sikdar J., Rai V. K. (2017). J. Anal. At. Spectrom..

[cit28] Liu X.-N., Hin R. C., Coath C. D., Bizimis M., Su L., Ionov D. A., Takazawa E., Brooker R., Elliott T. (2023). Geochim. Cosmochim. Acta.

[cit29] Poitrasson F. (2017). Rev. Mineral. Geochem..

[cit30] Goto K. (1956). J. Phys. Chem..

[cit31] Steele R. C. J., Coath C. D., Regelous M., Russell S., Elliott T. (2012). Astrophys. J..

[cit32] Zambardi T., Poitrasson F. (2011). Geostand. Geoanal. Res..

[cit33] Chen X., Lapen T. J., Chafetz H. S. (2017). Geostand. Geoanal. Res..

[cit34] Young E. D., Galy A., Nagahara H. (2002). Geochem. Cosmochim. Acta.

[cit35] Poitrasson F., Freydier R. (2005). Chem. Geol..

[cit36] Teng F. Z., Li W. Y., Ke S., Yang W., Liu S. A., Sedaghatpour F., Wang S. J., Huang K. J., Hu Y., Ling M. X. (2015). Geostand. Geoanal. Res..

[cit37] Teng F.-Z. (2017). Rev. Mineral. Geochem..

[cit38] Ng H. C., Hawkings J. R., Bertrand S., Summers B. A., Sieber M., Conway T. M., Freitas F. S., Ward J. P., Pryer H. V., Wadham J. L. (2022). Global Biogeochem. Cycles.

[cit39] Ng H. C., Cassarino L., Pickering R. A., Woodward E. M. S., Hammond S. J., Hendry K. R. (2020). Earth Planet. Sci. Lett..

[cit40] Chakrabarti R., Jacobsen S. B. (2010). Geochim. Cosmochim. Acta.

[cit41] Abraham K., Opfergelt S., Fripiat F., Cavagna A. J., De Jong J. T., Foley S. F., André L., Cardinal D. (2008). Geostand. Geoanal. Res..

[cit42] Pringle E. A., Savage P. S., Jackson M. G., Barrat J.-A., Moynier F. (2013). Astrophys. J..

[cit43] Oelze M., Schuessler J. A., von Blanckenburg F. (2016). J. Anal. At. Spectrom..

[cit44] Reynolds B. C., Aggarwal J., Andre L., Baxter D., Beucher C., Brzezinski M. A., Engström E., Georg R. B., Land M., Leng M. J. (2007). J. Anal.
At. Spectrom..

